# Effects of different iron sources on growth performance, intestinal morphology, development, and cell proliferation in weanling piglets

**DOI:** 10.1017/jns.2025.10054

**Published:** 2025-12-05

**Authors:** Ping Kang, Guolong Song, Jiajun Fan, Dianchao Gu, Qingqing Lv, Bingzhao Shi, Qingliang Chen, Kun Qin, Yanling Kuang, Dan Wang, Qiaoling Wen, Huiling Zhu, Yulan Liu

**Affiliations:** 1 Hubei Key Laboratory of Animal Nutrition and Feed Science, Wuhan Polytechnic University, Wuhan, Hubei, China; 2 Key Laboratory of Functional Aquafeed and Culture Environment Control, Fujian Dabeinong Huayou Aquatic Science and Technology Co. Ltd., Zhangzhou, Fujian, China; 3 DeBon Bio-Tech Co., Ltd., Hunan, China

**Keywords:** Growth performance, Iron, Intestinal development, Intestinal cell proliferation, Weanling piglets, ADFI, average daily feed intake, ADG, average daily gain, Bmi1, B cell-specific moloney murine ieukemia virus integration site 1, ChgA, chromogranin A, DM, dry matter, Fe-Gly, ferrous glycinate, F/G, feed: gain ratio, FeSO_4_, ferrous sulfate, H&E, hematoxylin and eosin, Lgr5, leucine rich repeat containing G protein-coupled receptor 5, Lyz, lysozyme, Muc2, mucin 2, Olfm, olfactomedin, PCNA, proliferating cell nuclear antigen, TIBC, total iron binding capacity, TSAT, transferrin saturation, ZO-1, zonula occluden-1

## Abstract

The current study aimed to investigate the effects of different iron sources on growth performance and small intestinal health in weaned piglets. Two hundred and forty piglets (*Duroc* × *Large White* × *Landrace*, 9.52 ± 1.60 kg, 40 ± 2 d) were assigned to four treatments including control group, a basal diet without iron supplemented in mineral premix; ferrous sulfate (FeSO_4_) group, 100 mg Fe/kg dry matter (DM); ferrous glycinate (Fe-Gly) group, 80 mg Fe/kg DM; amino acid-Fe(II)-chelator complexes group, 30 mg Fe/kg DM. There were four pens for each treatment, and each pen had fifteen piglets. The experiment lasted for 28 days. Compared to the control group, three iron sources increased average daily feed intake (*P* < 0.05). Fe-Gly and amino acid-Fe(II)-chelator complexes increased average daily gain (*P* < 0.05). Amino acid-Fe(II)-chelator complexes increased villus height in jejunum (*P* < 0.05). In addition, Fe-Gly increased Ki67 and leucine rich repeat containing G protein-coupled receptor 5 (Lgr5) mRNA expression in duodenum (*P* < 0.05). Amino acid-Fe(II)-chelator complexes increased claudin-1 mRNA expression, and both amino acid-Fe(II)-chelator complexes and Fe-Gly increased Lgr5 mRNA expression (*P* < 0.05) in jejunum. These results suggest that organic iron is more effective than FeSO_4_ in improving growth performance, and has a positive effect on intestinal health in weanling piglets.

## Graphical abstract

**Figure:**
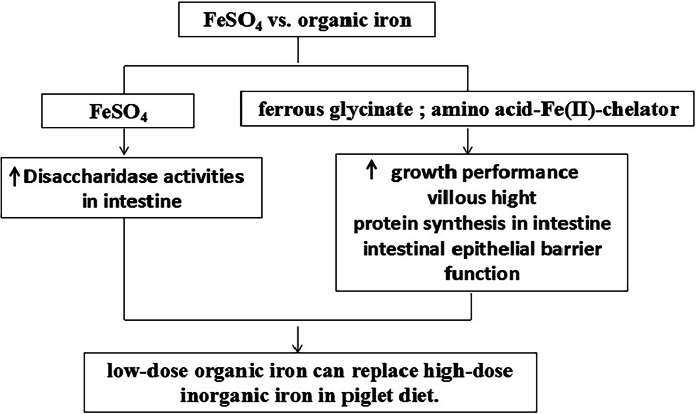

## Introduction

As one of the essential micronutrients, iron is required for growth, development, and many physiological processes. Intestinal iron absorption is an important process for maintaining body iron level within the optimal physiological range.^(1)^ Dietary iron is absorbed primarily in small intestine, and plays a crucial role in modulating intestinal development, epithelial maturation, and cell proliferation.^(2,3)^ Previous studies have found that iron promoted intestinal development and epithelial maturation in piglets.^(3,4)^

In animal production, ferrous sulfate (FeSO_4_) is usually supplemented as the standard inorganic iron source.^(5)^ However, ferrous salts can cause free-radical-mediated mucosal damage, even if they can rapidly correct the iron deficiency.^(6)^ In addition, FeSO_4_ has poor bioavailability,^(7)^ and unabsorbable iron is excreted in feces. Therefore, considering the environmental impact caused by pig manure iron, alternative iron should be evaluated in order to improve iron utilization.

Many studies have reported that organic iron has better bioavailability than inorganic iron.^(8,9)^ Zhuo *et al*.^(10)^ found that ferrous glycinate (Fe-Gly) was absorbed more efficiently and utilized faster than FeSO_4_. Lin *et al*.^(11)^ reported that Fe-Gly improved the bioavailability and antioxidant capacity of iron and reduced iron output of faeces. The mechanism may be due to the differences in absorption, transport and utilization processes.^(12)^ In addition, amino acid-Fe-chelator complexes have been proposed as a superior iron supplement for improving iron absorption and bioavailability.^(13)^ Therefore, the low-dose organic iron may have the same or higher efficiency than the high-dose inorganic iron in animal production.

Iron supplementation is necessary to prevent anaemia in piglets because of their special physiological condition.^(14,15)^ The current study aimed to investigate the effects of different iron sources on growth performance and small intestinal morphology, development, and proliferation in weaned piglets, and to explore whether the low-dose organic iron had the same or higher bioavailability on growth performance and intestine development than the high-dose inorganic iron in the weanling piglets.

## Materials and methods

### Experimental design

The animal use protocol for this research was approved by Wuhan Polytechnic University Institutional Animal Care and Use Committee (Wuhan, China). Two hundred and forty piglets (*Duroc* × *Large White* × *Landrace*, 9.52 ± 1.60 kg, 40 ± 2 d) were randomly assigned to four treatments: (1) control group, a basal diet without iron supplemented in mineral premix; (2) FeSO_4_ group (basal diet with iron compensation by FeSO_4_.H_2_O, FeSO_4_.H_2_O was bought from Lomon Corporation, purity 91.3%), 100 mg Fe/kg dry matter (DM); (3) Fe-Gly (Fe-Gly was kindly provided by DeBon Bio-Tech Co., Ltd. Fe≥17.0%) group, 80 mg Fe/kg DM; (4) amino acid-Fe(II)-chelator complexes (the complex was kindly provided by DeBon Bio-Tech Co., Ltd., Fe≥15.0%) group, 30 mg Fe/kg DM. There were four pens for each treatment, and each pen had fifteen piglets. The basal diet was corn-soybean diet and prepared to meet or exceed NRC^(16)^ nutrient requirement (Table 1). All piglets were allowed *ad libitum* access to water and feed during a 28-day experimental study. The ambient temperature was maintained at 22∼25 ℃ and the living environment was in accordance with animal welfare guidelines. The investigators monitored animals twice daily. Health was monitored by weight (once weekly), food and water intake, and general assessment of animal activity.

### Growth performance

Feed consumption was measured every day during the entire experimental time, and body weight was individually measured at the beginning of the trial, and at the end of experiment to calculate average daily gain (ADG), average daily feed intake (ADFI), and feed: gain ratio (F/G) in order to assess growth performance.

### Sample collection

On day 28, six healthy piglets were selected from each treatment group, with their body weights approximating the group mean. Then blood samples were collected via the jugular vein into 10-mL vacuum tubes on d 14 and d 28, respectively. The blood samples were centrifuged at 3000 × g for 10 min to collect serum, then frozen at −80℃ until further analysis. Blood haemoglobin was analyzed (#V-52D, diluted solution for animal blood cell analysis, #V-52DIFF, haemolytic agents for animal blood cell analysis, Shenzhen Mindray Animal Medical Technology Co., Ltd, Shenzhen, China) using a fully automated blood analyzer (ADVIA 2120i, Siemens, Germany). Serum iron concentration (#A039-1-1, serum iron assay kit), total iron binding capacity (TIBC, #A040-1-1, total iron binding capacity assay kit) were analyzed by the commercial assay kits (Nanjing Jiancheng Bioengineering Institute, Nanjing, China). Serum hepcidin level was determined by using a commercially available porcine ELISA assay kit (#RX500303P, pig hepcidin quantitative detection kit, Ruixin Biotechnology Co., Ltd, Quanzhou, China). After blood collection, pigs were slaughtered under anaesthesia with an intravenous injection of pentobarbital sodium (50 mg/kg BW). Duodenal and jejunal mucosa were collected, frozen in liquid nitrogen, and then stored in a freezer at –80°C for further analysis.

### Intestinal morphology

Approximately 0.5 cm of the second centimetre of each small intestinal section (duodenum and jejunum) was isolated, rinsed with sterile PBS, and fixed overnight with 10% formalin. Fixed tissues were embedded in paraffin, sectioned (5 μm), and stained with haematoxylin and eosin (H&E) for morphologic examinations. The lengths of villous height and crypt depth were measured by using Olympus software.

### DNA, RNA and protein contents in intestinal mucosa

The duodenum and jejunum mucosa were homogenized with a tissue homogenizer (PT-3100D, Kinematica, Switzerland). Protein concentration of mucosa homogenates was determined using a detergent-compatible protein assay (Bio-Rad Laboratories, Hercules, CA, USA) with bovine serum albumin as standards.^(17)^ Mucosa DNA content was evaluated by a fluorometric assay.^(18)^ Mucosa RNA content was measured according to the previous study.^(19)^ Briefly, the mucosa homogenate was mixed with perchloric acid and centrifuged. Then the precipitates were dissolved in potassium hydroxide (KOH). The absorbances of the supernatants at 232 and 260 nm were recorded and the micrograms of RNA per millilitre were calculated according to the formula.^(20)^

### Disaccharidase activities in intestine

The disaccharidase activities in intestinal mucosa homogenates were determined by the commercial assay kits (#A082-1-1, lactase assay kit; #A082-2-1, sucrase assay kit; #A082-3-1, maltase assay kit, Nanjing Jiancheng Bioengineering Institute, Nanjing, China). The absorbances were measured spectrophotometrically at 505 nm.

### Real-time PCR

The gene expression was measured by real-time PCR. Briefly, total RNA was isolated by the Trizol reagent (#9108, TaKaRa Biotechnology (Dalian) Co., Ltd., Dalian, China), and cDNA was synthesized using PrimeScript® RT reagent kit (#RR047A, TaKaRa Biotechnology (Dalian) Co., Ltd., Dalian, China). Real-time PCR assay was carried out on an ABI 7500 Real-Time PCR System (Applied Biosystems, Life Technologies) using a SYBR® Premix Ex TaqTM (Tli RNaseH Plus) qPCR kit (#RR420A, TaKaRa Biotechnology (Dalian) Co., Ltd., Dalian, China). The PCR cycling conditions were 95ºC × 30 s, followed by 40 cycles of 95ºC × 5 s and 60ºC × 34 s. The forward and reverse primers for the target genes were designed with Primer Premier 6.0 and synthesized by TaKaRa Biotechnology (Dalian, China, Table 2). The mRNA expression relative to housekeeping gene (GAPDH) was calculated according to the 2^−△△CT^ method.^(21)^

**Table 1.** Ingredient composition of diet (as fed basis)

Items	Content
Ingredients	%
Corn	55.50
Soybean meal (44% CP)	22.00
Wheat bran	3.00
Fish meal	5.50
Corn oil or fish oil	5.00
Soy protein concentrate	2.50
Milk-replacer powder	3.00
Limestone	0.70
Dicalcium phosphate	1.00
Salt	0.20
L-Lysine. HCl (78.8% Lysine)	0.27
Acidifier^a^	0.20
Butylated hydroquinone	0.05
Preservative^b^	0.05
Sweetener^c^	0.03
Vitamin and mineral premix^d^	1.00
Nutrient composition	
Digestible energy^e^ (MJ/kg)	14.0
Crude protein^f^	20.2
Ferrum^f^(mg/kg)	153.92

a A compound acidifier including lactic acid and phosphoric acid, provided by Wuhan Fanhua Biotechnology Company, Wuhan, China.

b A compound mould inhibitor including calcium propionate, fumaric acid, fumaric acid monoethyl ester and sodium diacetate, provided by Sichuan Minsheng Pharmaceutical Co., Ltd, Chengdu, China.

c A compound sweetener including saccharin sodium and disodium 5’-guanylate, provided by Wuhan Fanhua Biotechnology Company, Wuhan, China.

d The premix provided the following amounts per kilogram of complete diet: retinol acetate, 2700 μg; cholecalciferol, 62.5 μg; dl-α-tocopheryl acetate, 20 mg; menadione, 3 mg; vitamin B_12_, 18 μg; riboflavin, 4 mg; niacin, 40 mg; pantothenic acid, 15 mg; choline chloride, 400 mg; folic acid, 700 μg; thiamine, 1.5 mg; pyridoxine, 3 mg; biotin, 100 μg; Zn, 80 mg (ZnSO_4_·7H_2_O); Mn, 20 mg (MnSO_4_·5H_2_O); Cu, 25 mg (CuSO_4_·5H_2_O); I, 0.48 mg (KI); Se, 0.36 mg (Na_2_SeO_3_·5H_2_O).

e Calculated.

f Analyzed.

### Protein abundance analysis by Western blot

Quantification of protein expression in intestinal mucosa was performed as previously described.^(22,23)^ Briefly, extracted proteins were quantified, and separated by sodium dodecyl sulfate-polyacrylamide gel electrophoresis (SDS-PAGE). The separated proteins were then transferred to polyvinylidene difluoride membranes (Millipore). After blocking, membranes were incubated with primary antibodies at 4℃ overnight, followed by three 15-minute washes with PBS containing 0.1% Tween-20. The primary antibodies included rabbit anti-claudin-1 (#51-9000, Invitrogen), mouse anti-occludin (#ab31721, Abcam), mouse anti-zonula occluden-1 (ZO-1, #33-1500, invitrogen), and the secondary antibodies included goat anti-rabbit IgG-HRP (#ANT020, Wuhan Antejie Biotechnology Co., Ltd) and goat anti-mouse IgG-HRP (#ANT019, Wuhan Antejie Biotechnology Co., Ltd). The bands were analyzed by densitometry using GeneTools software (Syngene), and the abundance of each target protein was expressed as the target protein: β-actin ratio.

### Statistical analysis

The experimental data were analyzed by one-way ANOVA with SPSS 17.0 software. If the differences were significant, Duncan’s multiple comparisons were further used to determine the specific differences among experimental groups. All data were expressed as means ± SE. Differences were considered to be significant if *P* < 0.05.

## Results

### Growth performance

Growth performance is shown in Table 3. Compared with the control group, iron supplementation increased ADFI throughout the experimental period (*P* < 0.05). Diets supplemented with Fe-Gly or amino acid-Fe(II)-chelator complexes increased ADG (*P* < 0.05).

**Table 2.** Primer sequences used for real-time PCR^*^

Gene	Forward (5’ - 3’)	Reverse (5’ - 3’)	Product length (bp)	Accession numbers
Claudin-1	AGATTTACTCCTACGCTGGT	GCACCTCATCATCTTCCAT	249	NM_001244539.1
Occludin	ATCAACAAAGGCAACTCT	GCAGCAGCCATGTACTCT	157	NM_001163647.2
ZO-1	GAGTTTGATAGTGGCGTT	GTGGGAGGATGCTGTTGT	298	XM_021098856.1
PCNA	TACGCTAAGGGCAGAAGATAATG	CTGAGATCTCGGCATATACGTG	192	XM_003359883.1
ki67	GAAGATGTTGCCAGAATAGC	GAGGACCAGTGAATGAAGG	322	XM_021074506.1
Lgr5	CCTTGGCCCTGAACAAAATA	ATTTCTTTCCCAGGGAGTGG	111	XM_021090898.1
Olfm	GTCAGCAAACCGGCTATTGT	TGCCTTGGCCATAGGAAATA	226	XM_003482903.4
Bmi1	CGCTTGGCTCACATTCAT	GGACATCACAGATAGGACAAT	290	NM_001285971.1
Lyz	GGTCTATGATCGGTGCGAGT	AACTGCTTTGGGTGTCTTGC	214	NM_214392.2
ChgA	GGAGGAGAAGAAGGAGGAG	ACAGAGCCAGGACTACAG	187	NM_001164005.2
Muc2	GGCTGCTCATTGAGAGGAGT	ATGTTCCCGAACTCCAAGG	214	XM_021082584.1
Villin1	GTGGAGCAGGTGAAGTTC	CCGATGAGGTAGGTGTAGA	200	XM_001925167.6

* ZO-1, zonula occluden-1; PCNA, proliferating cell nuclear antigen; Lgr5, leucine rich repeat containing G protein-coupled receptor 5; Olfm, olfactomedin; Bmi1, B cell-specific moloney murine leukaemia virus integration site 1; Lyz, lysozyme; ChgA, chromogranin A ; Muc2, mucins.

### Blood haemoglobin and serum iron concentration, total iron binding capacity, transferrin saturation, and hepcidin concentration

As shown in Table 4, iron supplementation had no effect on blood haemoglobin concentration, serum iron concentration, TIBC, TSAT and hepcidin concentration in serum on d 14. On d 28, Fe-Gly and amino acid-Fe(II)-chelator complexes supplementation decreased serum iron concentration and TSAT (*P* < 0.05), and FeSO_4_ and Fe-Gly supplementation reduced TIBC (*P* < 0.05).

**Table 3.** Effects of different iron sources on growth performance in pigs during 1–28 days^*^

Item	Control	Ferrous sulfate	Ferrous glycinate	Amino acid-Fe(II)-chelator complexes	*P* value
Average daily gain (g)	322 ± 12^c^	349 ± 1^bc^	392 ± 15^a^	363 ± 10^ab^	0.011
Average daily feed intake (g)	575 ± 10^b^	613 ± 8^a^	636 ± 16^a^	612 ± 7^a^	0.026
Feed to gain ratio (g/g)	1.79 ± 0.04	1.76 ± 0.02	1.62 ± 0.02	1.69 ± 0.05	0.064

* Values are means ±SE, *n* = 4 per treatment.

a-c Values in the same row with different superscript letters are significantly different (*P* < 0.05).

### Intestinal morphology

As shown in Table 5, regardless of iron sources, iron supplementation decreased villus height, and FeSO_4_ and amino acid-Fe(II)-chelator complexes supplementation reduced crypt depth in duodenum (*P* < 0.05). However, amino acid-Fe(II)-chelator complexes supplementation increased villus height in jejunum (*P* < 0.05). Both FeSO_4_ and Fe-Gly decreased crypt depth in jejunum (*P* < 0.05).

**Table 4.** Effects of different iron sources on blood haemoglobin concentration, serum iron concentration, total iron-binding capacity (TIBC), transferrin saturation and hepcidin concentration in pigs at 14 and 28 days^*^

Items	Control	Ferrous sulfate	Ferrous glycinate	Amino acid-Fe(II)-chelator complexes	*P Value*
14 d					
Haemoglobin (g/L)	66.00 ± 2.39	57.86 ± 2.84	66.20 ± 1.77	58.50 ± 4.92	0.218
Serum iron concentration (μmol/L)	30.17 ± 1.11	42.61 ± 4.11	38.00 ± 4.52	42.67 ± 6.24	0.182
Total iron binding capacity (μmol/L)	126.09 ± 8.85	124.34 ± 7.96	134.12 ± 13.23	134.64 ± 4.90	0.800
Transferrin saturation (%)	24.29 ± 1.15	34.67 ± 3.33	29.03 ± 3.93	31.45 ± 4.02	0.191
Hepcidin (ng/mL)	49.35 ± 3.16	46.06 ± 3.88	44.17 ± 1.61	49.50 ± 5.24	0.683
28 d					
Haemoglobin (g/L)	53.80 ± 8.79	56.40 ± 5.92	58.60 ± 11.14	54.00 ± 8.79	0.977
Serum iron concentration (μmol/L)	47.64 ± 1.98^a^	48.69 ± 3.29^a^	31.17 ± 3.34^b^	37.80 ± 2.73^b^	0.001
Total iron binding capacity (μmol/L)	124.01 ± 3.66^a^	102.25 ± 6.96^b^	105.57 ± 5.24^b^	109.91 ± 5.30^ab^	0.049
Transferrin saturation (%)	40.23 ± 3.64^ab^	47.70 ± 1.12^a^	29.60 ± 2.35^c^	34.77 ± 3.00^bc^	0.001
Hepcidin (ng/mL)	42.96 ± 3.70	48.50 ± 7.55	54.64 ± 3.79	64.20 ± 6.60	0.071

* Values are means ±SE, *n* = 6 per treatment.

a-c Values in the same row with different superscript letters are significantly different (*P* < 0.05).

### Protein, RNA and DNA levels

Protein, RNA and DNA levels in intestine are shown in Table 6. Amino acid-Fe(II)-chelator complexes supplementation increased protein to DNA ratio in duodenum (*P* < 0.05), and increased protein content, RNA to DNA ratio, and protein to DNA ratio in jejunum (*P* < 0.05).

**Table 5.** Effects of different iron sources on intestinal morphology in pigs at 28 d^*^

Item	Control	Ferrous sulfate	Ferrous glycinate	Amino acid-Fe(II)-chelator complexes	*P* value
Duodenum					
Villus height (μm)	420 ± 6^a^	351 ± 12^b^	376 ± 7^b^	350 ± 16^b^	0.001
Crypt depth (μm)	200 ± 3^a^	174 ± 5^c^	193 ± 6^ab^	175 ± 10^bc^	0.016
Villus height/Crypt depth	2.10 ± 0.05	2.02 ± 0.06	1.95 ± 0.04	2.01 ± 0.08	0.389
Jejunum					
Villus height (μm)	383 ± 11^b^	340 ± 7^b^	393 ± 23^b^	471 ± 25^a^	0.001
Crypt depth (μm)	197 ± 4^a^	145 ± 9^b^	162 ± 3^b^	194 ± 14^a^	0.001
Villus height/Crypt depth	1.95 ± 0.04^b^	2.37 ± 0.12^a^	2.42 ± 0.12^a^	2.47 ± 0.14^a^	0.014

* Values are means ±SE, *n* = 6 per treatment.

a-c Values in the same row with different superscript letters are significantly different (*P* < 0.05).

### Disaccharidase activities in intestine

Disaccharidase activities in intestine are shown in Table 7. Amino acid-Fe(II)-chelator complexes decreased maltase activity in both duodenum and jejunum, and Fe-Gly increased sucrase activity in duodenum (*P* < 0.05). FeSO_4_ increased lactase and sucrase activities in jejunum (*P* < 0.05).

**Table 6.** Effects of different iron sources on the protein, DNA and RNA contents in intestine of pigs at 28 d^*^

Items	Control	Ferrous sulfate	Ferrous glycinate	Amino acid-Fe(II)-chelator complexes	*P Value*
Duodenum					
Protein (mg/g)	92.43 ± 3.98	85.95 ± 3.23	90.22 ± 3.68	89.28 ± 2.01	0.585
RNA/DNA (μg/μg)	3.53 ± 0.27	3.52 ± 0.18	4.04 ± 0.24	3.99 ± 0.16	0.199
Protein/DNA (mg/μg)	0.145 ± 0.006^b^	0.146 ± 0.005^b^	0.159 ± 0.007^ab^	0.166 ± 0.003^a^	0.037
Jejunum					
Protein (mg/g)	88.53 ± 2.24^b^	97.62 ± 3.78^b^	86.61 ± 4.77^b^	108.85 ± 3.87^a^	0.002
RNA/DNA (μg/ug)	3.50 ± 0.13^b^	4.02 ± 0.19^ab^	3.25 ± 0.42^b^	4.44 ± 0.27^a^	0.026
Protein/DNA (mg/μg)	0.157 ± 0.004^b^	0.188 ± 0.004^a^	0.147 ± 0.016^b^	0.210 ± 0.009^a^	< 0.001

* Values are means ±SE, *n* = 6 per treatment.

a, b Values in the same row with different superscript letters are significantly different (*P* < 0.05).

### mRNA expression of intestinal epithelial barrier function, and cell proliferation related genes

As shown in Table 8, compared with control group, both FeSO_4_ and Fe-Gly supplementation increased Ki67 mRNA expression, and Fe-Gly supplementation increased leucine rich repeat containing G protein-coupled receptor 5 (Lgr5) mRNA expression in duodenum (*P* < 0.05). In jejunum, amino acid-Fe(II)-chelator complexes increased claudin-1 mRNA expression (*P* < 0.05), and both amino acid-Fe(II)-chelator complexes and Fe-Gly increased Lgr5 mRNA expression (*P* < 0.05), Fe-Gly increased Lysozyme (Lyz) mRNA expression, and FeSO_4_ and amino acid-Fe(II)-chelator complexes increased Villin mRNA expression (*P* < 0.05).

**Table 7.** Effects of different iron sources on the disaccharidase activities in intestine of pigs at 28 d^*^

Item	Control	Ferrous sulfate	Ferrous glycinate	Amino acid-Fe(II)-chelator complexes	*P* value
Duodenum					
Maltase activity (U/mg protein)	45.27 ± 4.98^ab^	50.57 ± 4.31^a^	37.71 ± 3.25^bc^	29.19 ± 2.76^c^	0.006
Lactase activity (U/mg protein)	8.23 ± 1.37	8.62 ± 1.53	6.80 ± 1.77	6.40 ± 1.43	0.689
Sucrase activity (U/mg protein)	4.89 ± 0.77^b^	4.31 ± 0.75^b^	8.07 ± 0.81^a^	4.81 ± 1.19^b^	0.030
Jejunum					
Maltase activity (U/mg protein)	66.27 ± 4.37^a^	64.83 ± 5.01^a^	67.92 ± 4.44^a^	50.29 ± 2.44^b^	0.027
Lactase activity (U/mg protein)	11.49 ± 1.56^b^	21.37 ± 1.59^a^	10.18 ± 1.79^b^	7.52 ± 1.21^b^	< 0.001
Sucrase activity (U/mg protein)	11.21 ± 0.88^b^	15.17 ± 1.07^a^	11.69 ± 0.89^b^	10.58 ± 1.41^b^	0.032

* Values are means ±SE, *n* = 6 per treatment.

a-c Values in the same row with different superscript letters are significantly different (*P* < 0.05).

**Table 8.** Effects of different iron sources on intestinal epithelial barrier function and proliferation-related genes expression in pigs at 28 d^*^

Item	Control	Ferrous sulfate	Ferrous glycinate	Amino acid-Fe(II)-chelator complexes	*P* value
Duodenum					
Claudin-1	1.00 ± 0.13	0.97 ± 0.15	0.78 ± 0.10	0.68 ± 0.04	0.176
Occludin	1.00 ± 0.16	1.11 ± 0.07	1.06 ± 0.07	1.25 ± 0.14	0.502
ZO-1	1.00 ± 0.10	1.30 ± 0.12	1.21 ± 0.12	1.27 ± 0.13	0.308
PCNA	1.00 ± 0.14	0.69 ± 0.10	0.95 ± 0.16	0.88 ± 0.12	0.384
Ki67	1.00 ± 0.09^ab^	0.82 ± 0.07^b^	0.87 ± 0.09^b^	1.23 ± 0.14^a^	0.046
Lgr5	1.00 ± 0.16^bc^	1.28 ± 0.29^b^	2.13 ± 0.22^a^	0.58 ± 0.08^c^	< 0.001
Olfm4	1.00 ± 0.11	1.06 ± 0.23	1.06 ± 0.16	0.72 ± 0.15	0.442
Bmi1	1.00 ± 0.12	1.09 ± 0.16	1.25 ± 0.11	1.07 ± 0.07	0.534
Lyz	1.00 ± 0.24	0.41 ± 0.04	1.99 ± 0.67	0.95 ± 0.47	0.101
ChgA	1.00 ± 0.01	1.34 ± 0.16	1.44 ± 0.22	1.20 ± 0.18	0.279
Muc2	1.00 ± 0.18	0.89 ± 0.09	0.97 ± 0.11	0.85 ± 0.06	0.777
Villin	1.00 ± 0.03	1.05 ± 0.08	0.96 ± 0.09	1.26 ± 0.18	0.261
Jejunum					
Claudin-1	1.00 ± 0.10^c^	1.87 ± 0.37^ab^	1.10 ± 0.14^bc^	2.11 ± 0.35^a^	0.019
Occludin	1.00 ± 0.12	1.15 ± 0.10	0.98 ± 0.11	1.17 ± 0.11	0.515
ZO-1	1.00 ± 0.13^b^	1.18 ± 0.14^ab^	1.01 ± 0.08^b^	1.42 ± 0.11^a^	0.069
PCNA	1.00 ± 0.06	0.84 ± 0.17	1.26 ± 0.07	1.07 ± 0.11	0.108
Ki67	1.00 ± 0.05	1.09 ± 0.16	1.16 ± 0.12	1.38 ± 0.17	0.255
Lgr5	1.00 ± 0.06^b^	1.10 ± 0.20^b^	2.11 ± 0.19^a^	2.57 ± 0.45^a^	0.001
Olfm4	1.00 ± 0.15	0.70 ± 0.16	1.16 ± 0.15	0.93 ± 0.15	0.218
Bmi1	1.00 ± 0.02^b^	0.91 ± 0.10^b^	1.06 ± 0.14^ab^	1.37 ± 0.15^a^	0.058
Lyz	1.00 ± 0.09^b^	0.74 ± 0.18^b^	1.74 ± 0.16^a^	0.96 ± 0.26^b^	0.006
ChgA	1.00 ± 0.05	0.84 ± 0.12	1.30 ± 0.19	1.14 ± 0.09	0.095
Muc2	1.00 ± 0.12	0.75 ± 0.09	0.70 ± 0.11	0.78 ± 0.08	0.178
Villin	1.00 ± 0.03^b^	1.40 ± 0.14^a^	1.03 ± 0.06^b^	1.52 ± 0.18^a^	0.013

* ZO-1, zonula occluden-1; PCNA, proliferating cell nuclear antigen; Lgr5, leucine rich repeat containing G protein-coupled receptor 5; Olfm, olfactomedin; Bmi1, B cell-specific moloney murine leukaemia virus integration site 1; Lyz, lysozyme; ChgA, chromogranin A; Muc2, mucins

Values are means ±SE, *n* = 6 per treatment.

a-c Values in the same row with different superscript letters are significantly different (*P* < 0.05).

### Protein expression of claudin-1, occludin, and ZO-1 in intestine

**Figure 1.**
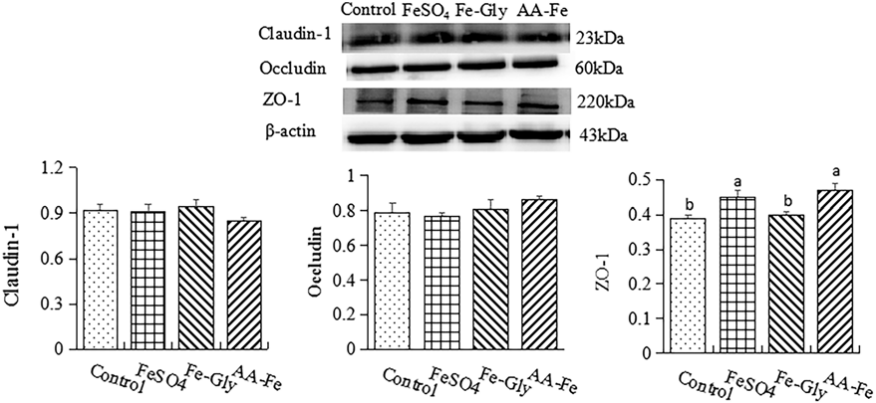
The effects of different iron sources on protein expression of claudin-1, occludin, and ZO-1 in duodenum in the weaned piglets. Values were means (*n* = 6), with their standard errors represented by vertical bars. () Control group, a basal diet without iron supplemented in mineral premix; () ferrous sulfate (FeSO_4_) group, 100 mg Fe/kg dry matter (DM); () ferrous glycinate (Fe-Gly) group, 80 mg Fe/kg DM; () amino acid-Fe(II)-chelator complexes group, 30 mg Fe/kg DM.

**Figure 2.**
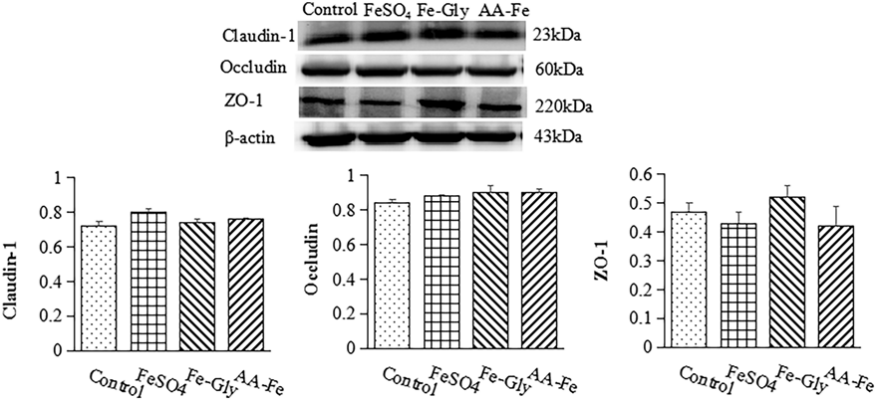
The effects of different iron sources on protein expression of claudin-1, occludin, and ZO-1 in jejunum in the weaned piglets. Values were means (*n* = 6), with their standard errors represented by vertical bars. () Control group, a basal diet without iron supplemented in mineral premix; () ferrous sulfate (FeSO_4_) group, 100 mg Fe/kg dry matter (DM); () ferrous glycinate (Fe-Gly) group, 80 mg Fe/kg DM; () amino acid-Fe(II)-chelator complexes group, 30 mg Fe/kg DM.

As shown in Figure 1 and 2, different iron sources supplementation had no effect on protein expression of claudin-1 and occludin in duodenum. FeSO_4_ and amino acid-Fe(II)-chelator complexes increased protein expression of ZO-1 in duodenum (*P* < 0.05). However, protein expression of claudin-1, occludin, and ZO-1 in jejunum had no difference among these treatments.

## Discussion

Iron is an essential micronutrient and required for growth, development, and many physiological processes. Increasing dietary iron can improve growth performance in piglets.^(24)^ In this study, the iron content in the basal diet was 153.92 mg/kg. However, animals exhibit limited ability to utilize dietary iron.^(25)^ Consequently, the iron in basal diet is typically negligible in practical production. FeSO_4_ is usually supplemented as the standard inorganic iron source,^(6)^ and its supplementation exhibits a dose-dependent increase in ADG in piglets^(26)^ and growing-finishing pigs.^(27)^ Previous studies have demonstrated that organic iron exhibits superior bioavailability compared to inorganic iron.^(9,10,28)^ Furthermore, amino acid-Fe-chelator complexes have been proposed as superior iron supplements due to their potentially higher biological utilization efficiency. Therefore, in this study, two types of organic iron compounds with different doses were selected to investigate: (1) whether low-dose organic iron could achieve comparable or superior efficacy to high-dose inorganic iron, and (2) whether low-dose amino acid-Fe-chelator complexes could exhibit bioavailability equivalent to high-dose Fe-Gly. In this study, we found both Fe-Gly and amino acid-Fe(II)-chelator complexes increased ADG and tended to decrease F/G. However, no significant differences were observed between two organic iron sources. Our results suggest that organic iron sources including both Fe-Gly and amino acid-Fe(II)-chelator complexes were more effective in improving growth performance in weanling pigs compared to inorganic iron. Notably, lower-dose amino acid-Fe(II)-chelator complexes achieved comparable efficacy to Fe-Gly in enhancing growth performance.

TIBC and TSAT are important biochemical markers of body iron status. Low TSAT level is an indicator of iron deficiency.^(29,30)^ However, elevated TSAT can damage cells and tissues through oxidative stress.^(31)^ Zhou *et al*.^(12)^ found that Fe-Gly increased TSAT in SD rats. However, inconsistent with this report, we found that Fe-Gly supplementation decreased TSAT and serum iron concentration compared to FeSO_4_. Moreover, we found Fe-Gly and amino acid-Fe(II)-chelator complexes decreased serum iron concentration, but this result might not mean iron deficiency. Nemeth and Ganz^(32)^ reported serum iron concentration was low despite adequate iron stores. The results of growth performance in this study also imply this inference.

Hepcidin is the central regulator of iron homeostasis in the body. The production of hepcidin in hepatocytes can be greatly stimulated by plasma iron and iron stores.^(33)^ Then, the increased hepcidin can inversely lead to decreased iron absorption and release of iron from stores. In this study, in consistent with the results of the decreased serum iron concentration, organic iron had a tendency to increase serum hepcidin concentration, which indicated that organic iron had better bioavailability to be absorbed easily, then stimulated hepcidin production to decrease serum iron concentration.

Villus height and crypt depth is closely related to primary function of digestion and absorption of nutrients in small intestine.^(34)^ Moderate FeSO_4_ can increase duodenal villus height and the colonic crypt depth.^(27)^ Zhuo *et al*.^(6)^ found diet supplement with iron significantly increased villus height, and Fe-Gly exhibited better bioavailability than FeSO_4_. However, contrary to the above results, we found that villus height in duodenum was higher in control group than other groups. Wayhs *et al.* ^(35)^ reported that iron deficiency had a compensatory intestinal mechanism to increase intestinal villus height to improve iron absorption. Therefore, the higher villus height in the control group might result from the iron deficiency, which stimulated the compensatory intestinal mechanism to increase iron absorption.

Iron can stimulate DNA and protein synthesis.^(36,37)^ Protein/DNA ratio can be a measure of protein concentration^(38)^ and cell size.^(39)^ RNA/DNA ratio provides a measure of synthetic capacity of cell and indicates the capacity of protein synthesis.^(40)^ Higher RNA/DNA ratio shows the increased ability to synthesize intracellular RNA and protein.^(41)^ Higashida *et al*.^(36)^ found iron was essential to protein synthesis in skeletal muscle, and its deficiency was associated with muscle weakness. In addition, impaired protein synthesis resulting from iron deficiency in pups limited their ability to produce antibodies. Our results showed that amino acid-Fe(II)-chelator complexes had a greater efficacy in increasing protein synthesis in intestine. However, Yamauchi *et al.* ^(43)^. found that protein deficiency was associated with decreased intestinal villous height, whereas in our study, amino acid-Fe(II)-chelator complexes increased protein synthesis but reduced villus height. The mechanism requires further investigation.

Disaccharidases are crucial for the intestinal function. Both iron deficiency and iron excess decrease disaccharidase activities.^(44,45)^ Many studies reported that iron deficiency decreased the activity of disaccharidases,^(44,46)^ especially lactase,^(47,48)^ which might result from the reduced ability to synthesize lactase in enterocytes.^(49)^ The present study found that amino acid-Fe(II)-chelator complexes reduced maltase activity in intestine. However, the performance data, as well as the increased protein/DNA ratio suggested that iron supplemented by amino acid-Fe(II)-chelator complexes was enough in this study.

Claudin-1, occludin, and ZO-1 are tight junction proteins, which can maintain the intestinal epithelial barrier function. Iron can enhance intestinal barrier function by increasing the colonic expressions of occludin and claudin-1.^(50)^ Sun *et al*.^(51)^ found Fe-Gly supplementation increased the abundance of tight-junction protein ZO-1 in jejunum in weaned piglets. However, overload ferric citrate impairs intestinal barrier function evidenced by the reduced tight junction proteins.^(52)^ Wu *et al.* ^(53)^ also found iron overload decreased the levels of ZO-1 and Occludin. In this study, the increased mRNA expression of claudin-1 and ZO-1 in jejunum after amino acid-Fe(II)-chelator complexes supplemented suggested that it could have a positive role to form a barrier in small intestine and prevent pathogen invasion. In our study, the protective effects of amino acid-Fe(II)-chelator complex supplementation on intestinal integrity may be closely related to protein synthesis capacity in the intestine.

Ki-67 protein is a cell proliferation-associated nuclear marker.^(54)^ The number of Ki67-positive cells is usually in line with the proliferation of gastrointestinal epithelial cells.^(55)^ Lgr5 is a marker of active stem cells in the small intestine and colon.^(56)^ B cell-specific moloney murine leukaemia virus integration site 1 (Bmi1) is adult stem cell gene. Precious study found heat stress inhibited the intestinal epithelial cell proliferation and stem cell expansion by down-regulating the expression of Ki67, Lgr5, and Bmi1.^(57)^ Zhou *et al*.^(55)^ found FeSO_4_ decreased the expression of Lgr5 in jejunum. However, our results showed that Fe-Gly had a better effect on activating stem cells in the small intestine. In addition, Lyz can protect intestinal epithelium against bacterial infection^(58)^ and enhance intestinal functions and gut microflora of piglets.^(59)^ In swine feed, it can be supplemented as the alternative to growth-promoting subtherapeutic antibiotic.^(60)^ Villin is the marker of absorptive cells,^(61)^ its expression can assess recovery of the intestinal absorptive surface area.^(62)^ In this study, we found that organic iron improved and maintained jejunal epithelial barrier and absorption function, as evidenced by increased mRNA expressions of Lyz, Villin, claudin-1, and ZO-1 in the jejunum. These results were consistent with the imoroved growth performance observed in piglets, suggesting that this enhancement may result from enhanced intestinal absorption capacity and barrier function.

## Conclusion

In summary, organic iron is more effective than FeSO_4_ in improving performance, and has a positive effect on intestinal health in the weanling piglets. Low-dose organic iron may replace high-dose inorganic iron in piglet diet.
